# Diagnostic and prognostic value of serum C-reactive protein in heart failure with preserved ejection fraction: a systematic review and meta-analysis

**DOI:** 10.1007/s10741-020-09927-x

**Published:** 2020-02-06

**Authors:** Ishan Lakhani, Michelle Vangi Wong, Joshua Kai Fung Hung, Mengqi Gong, Khalid Bin Waleed, Yunlong Xia, Sharen Lee, Leonardo Roever, Tong Liu, Gary Tse, Keith Sai Kit Leung, Ka Hou Christien Li

**Affiliations:** 1 Li Ka Shing Institute of Health Sciences, Faculty of Medicine, Chinese University of Hong Kong, Hong Kong, SAR, People’s Republic of China; 2 Tianjin Key Laboratory of Ionic-Molecular Function of Cardiovascular disease, Department of Cardiology, Tianjin Institute of Cardiology, Second Hospital of Tianjin Medical University, Tianjin, 300211 People’s Republic of China; 3 Department of Cardiovascular Medicine, The First Affiliated Hospital of Dalian Medical University, Dalian, China; 4 Department of Clinical Research, Federal University of Uberlândia, Uberlândia, Brazil; 5 Xiamen Cardiovascular Hospital, Xiamen University, Xiamen, Fujian People’s Republic of China; 6 Aston Medical School, Aston University, Birmingham, UK; 7 Wirral University Teaching Hospital NHS Foundation Trust, Arrowe Park Hospital, Arrowe Park Rd, Birkenhead, Wirral CH49 5PE UK

**Keywords:** C-reactive protein, Diastolic heart failure, HFpEF, Meta-analysis

## Abstract

Heart failure (HF) is a major epidemic with rising morbidity and mortality rates that encumber global healthcare systems. While some studies have demonstrated the value of CRP in predicting (i) the development of HFpEF and (ii) long-term clinical outcomes in HFpEF patients, others have shown no such correlation. As a result, we conducted the following systematic review and meta-analysis to assess both the diagnostic and prognostic role of CRP in HFpEF. PubMed and Embase were searched for studies that assess the relationship between CRP and HFpEF using the following search terms: (((C-reactive protein) AND ((preserved ejection fraction) OR (diastolic heart failure))). The search period was from the start of database to August 6, 2019, with no language restrictions. A total of 312 and 233 studies were obtained from PubMed and Embase respectively, from which 19 studies were included. Our meta-analysis demonstrated the value of a high CRP in predicting the development of not only new onset HFpEF (HR: 1.08; 95% CI: 1.00–1.16; *P* = 0.04; *I*^2^ = 22%), but also an increased risk of cardiovascular mortality when used as a categorical (HR: 2.52; 95% CI: 1.61–3.96; *P* < 0.0001; *I*^2^ = 19%) or a continuous variable (HR: 1.24; 95% CI: 1.04–1.47; *P* = 0.01; *I*^2^ = 28%), as well as all-cause mortality when used as a categorical (HR: 1.78; 95% CI: 1.53–2.06; *P* < 0.00001; *I*^2^ = 0%) or a continuous variable: (HR: 1.06; 95% CI: 1.02–1.06; *P* = 0.003; *I*^2^ = 61%) in HFpEF patients. CRP can be used as a biomarker to predict the development of HFpEF and long-term clinical outcomes in HFpEF patients, in turn justifying its use as a simple, accessible parameter to guide clinical management in this patient population. However, more prospective studies are still required to not only explore the utility and dynamicity of CRP in HFpEF but also to determine whether risk stratification algorithms incorporating CRP actually provide a material benefit in improving patient prognosis.

**Electronic supplementary material:**

The online version of this article (10.1007/s10741-020-09927-x) contains supplementary material, which is available to authorized users.

## Introduction

Heart failure (HF) is a major epidemic with rising morbidity and mortality rates that encumber global healthcare systems. The 2016 European Society of Cardiology Guidelines denotes three classes of HF, stratified primarily according to ejection fraction: (i) HF with reduced ejection fraction (HFrEF), wherein EF in less than 40%; (ii) HF with midrange ejection fraction (HFmrEF), wherein EF is between 40 and 50%; (iii) HF with preserved ejection fraction (HFpEF), wherein EF is greater than 50% [1]. Although an abundance of evidence exists detailing the epidemiology, pathophysiology, and optimal treatment strategies for HFrEF, there much unknown pertaining to its counterpart, HFpEF.

The pathogenesis of both HFrEF and HFpEF differ, and as such, therapies proven to be effective for HFrEF have often failed to yield the same favorable outcomes in HFpEF cohorts. One of the suggested mechanisms contributing to the development of HFpEF is a systemic inflammatory state that adversely affects cardiomyocyte function on a molecular level. This inflammatory milieu is characterized by an elevation in the serum levels of various biomarkers [2], one of which is C-reactive protein (CRP): an acute phase protein produced by hepatocytes in a reactive response to inflammation [3].

Many investigations have analyzed the use of CRP as a biomarker to predict not only the development of HFpEF but also long-term clinical outcomes that may occur in HFpEF patients. However, while some studies have demonstrated a relationship between CRP and HFpEF, others have shown no such correlation. As a result, we conducted the following systematic review and meta-analysis to assess both the diagnostic and prognostic role of CRP in HFpEF.

## Methods

### Search strategy, inclusion, and exclusion criteria

This systematic review and meta-analysis was performed according to the Preferred Reporting Items for Systematic Reviews and Meta-Analyses (PRISMA) statement [4]. PubMed and Embase were searched up to August 6, 2019, with no language restrictions, for studies assessing the relationship between CRP and HFpEF development as well as outcomes in HFpEF patients. The following search terms were used: (C-reactive protein) AND ((preserved ejection fraction) OR (diastolic heart failure)). The inclusion criteria was as follows: (i) the study was a prospective or retrospective cohort study in humans and (ii) hazard ratios for risk of new onset HFpEF as well as prognostic outcomes including cardiovascular (CV) mortality, long-term CV outcomes, and all-cause mortality were reported in the published data.

The Newcastle–Ottawa Quality Assessment Scale (NOS) was used for quality assessment of the included studies. The NOS point score system evaluated the categories of study participant selection, comparability of the results, and quality of the outcomes. The following characteristics were assessed: (a) representativeness of the exposed cohort, (b) selection of the non-exposed cohort, (c) ascertainment of exposure, (d) demonstration that outcome of interest was not present at the start of study, (e) comparability of cohorts based on study design or analysis, (f) assessment of outcomes; (g) follow-up periods that were sufficiently long for outcomes to occur, and (h) adequacy of follow-up of cohorts. This scale ranged from zero to nine stars, which indicated that studies were graded as poor quality if the score was < 5, fair if the score was 5 to 7, and good if the score was > 8.

### Data extraction and statistical analysis

Data from the different studies were entered in pre-designed spreadsheets using Microsoft Excel. All abstracts were retrieved as complete manuscripts and assessed against the inclusion criteria. The data extracted include: (i) publication details: last name of first author, publication year, and locations; (ii) study design; (iii) follow-up duration; (iv) endpoint(s); (v) quality score; and (vii) characteristics of the population including sample size, gender, age, and number of subjects. Two reviewers (IL and KL) reviewed each included study independently. Disagreements were resolved by adjudication with input from a third reviewer (GT).

Statistical analysis was performed using the Comprehensive Meta-Analysis Software (Version 3). Heterogeneity between studies was determined using Cochran’s *Q* value, the weighted sum of squared differences between individual study effects and the pooled effect across studies, and the *I*^*2*^ statistic determined from the standard chi-square test, which describes the proportion of total variance-explained heterogeneity. *I*^*2*^ > 50% was considered to reflect significant statistical heterogeneity. A fixed effects model was used if *I*^*2*^ < 50%; otherwise, the random effects model using the inverse variance heterogeneity method was used. To identify the source of the heterogeneity, a sensitivity analysis using the leave-one-out method was performed. Funnel plots, Begg and Mazumdar rank correlation test, and Egger’s test were also used to assess for possible publication bias.

## Results

**Fig. 1:**
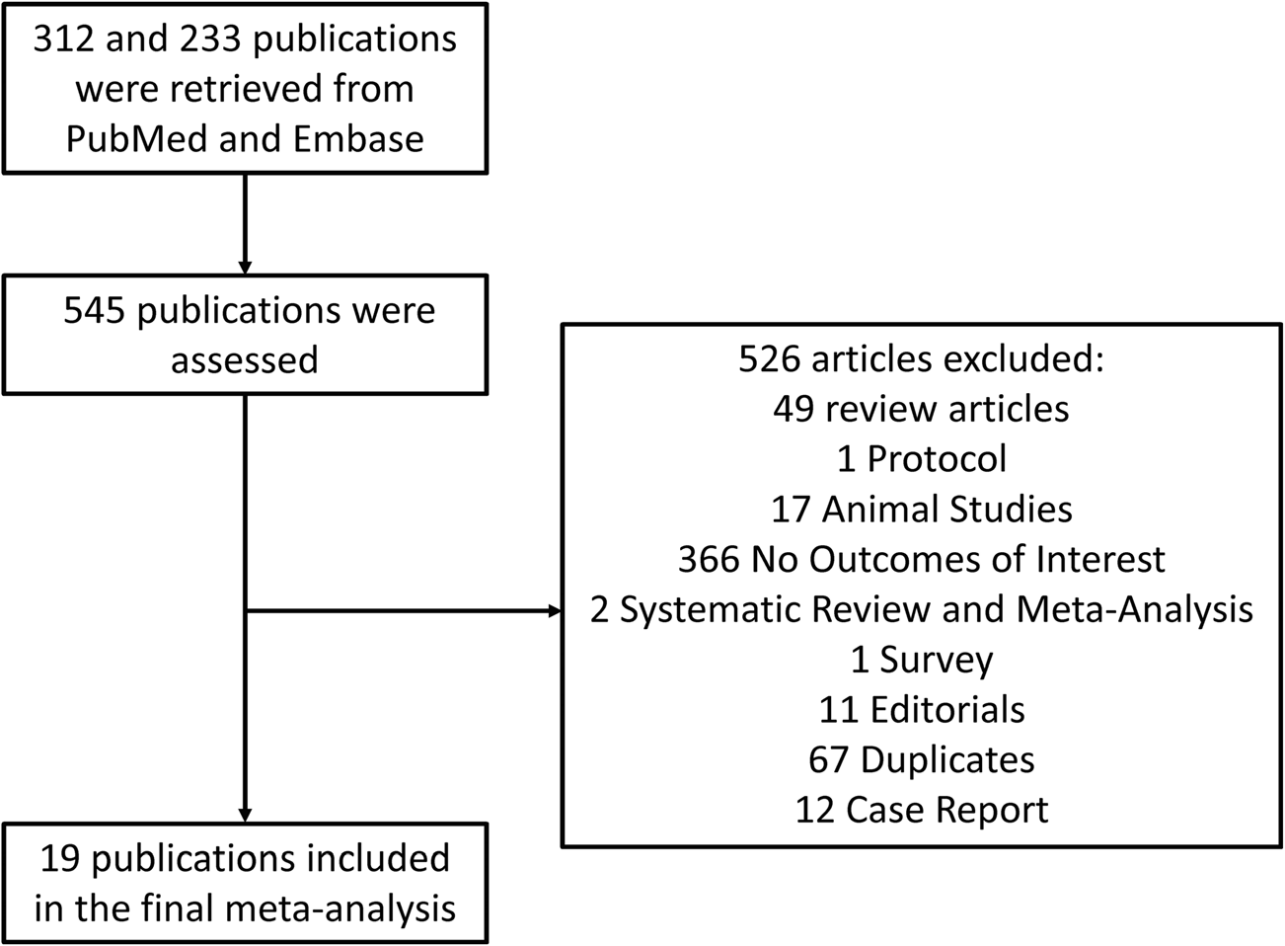
Study identification and selection process from PubMed and Embase

Figure 1 demonstrated the study identification and selection process. Three hundred and twelve and 233 studies were obtained from PubMed and Embase respectively, from which a total of 19 studies satisfied the inclusion criteria. The details of the NOS quality assessment for the included cohort studies are shown in Supplementary Table 1. Baseline characteristics of these studies and of the study populations are shown in Table 1. A total of 51,196 patients with a mean age of 61.1 ± 13.9 years were included. Fifty percent of the population was male, and the mean follow-up period was 120 months.

**Table 1:** Baseline characteristics of the 19 studies included in this meta-analysis

Study	Sample size (*n*)	No. of males	Age	SD	Outcome	Univariate (*U*)/multivariate (*M*)	Variables included	Study type	Follow-up duration (months)
Brouwers 2014	8569	4267	49	12.7	New onset HFpEF risk	*M*	Age, sex, BMI, smoking status, SBP, AF, plasma glucose, and total cholesterol levels	R	150
De Boer CHS 2018	5277	2239	73	6		*M*	Age, sex, race/ethnicity, previous MI, BMI, HT treatment, SBP, smoking status, presence of left ventricular hypertrophy or left bundle branch block, and diabetes.	R	144
De Boer FHS 2018	3431	1605	59	10		*M*			
De Boer MESA 2018	6679	3158	62	10		*M*			
De Boer PREVEND 2018	7369	3367	49	12		*M*			
Kalogeropoulos 2009	2610	1260	73.6	2.9		*M*	Controlling for baseline characteristics	R	113
Silverman 2016	6742	3178	68.5	9.1		*M*	Significant variables in univariate analysis	R	134
Tromp 2017	460	172	70.6	11.1		*U*	N/A	R	18
AlBadri 2017	390	0	56	11	All-cause mortality	*M*	Age, smoking history, diabetes, and statins	P	72
Aramburu-Bodas 2015	354	110	74.7	8.6	All-cause mortality (categorical); cutoff: ≥ 14.25 mg/l	*U*	N/A	P	12
Chen 2013	170	139	63.6	9.8	Long-term CV events (categorical); cutoff: ≥ 2.9 mg/l	*M*	Not listed	R	120
Hirata 2017	424	263	70.3	8.9	Long-term CV events (continuous)	*U*	N/A	P	20
Imai 2017	278	115	79.3	12.1	All-cause mortality (continuous)	*U*	N/A	P	36
Koller 2014	459	291	67.9 (median)	N/A	1. All-cause mortality (continuous and categorical); cutoff: > 13.8 mg/l 2. CV mortality (continuous and categorical); cutoff: > 13.8 mg/l	*M*	Age, sex, NYHA classification, NT-proBNP, eGFR, smoking, HT, CAD, diabetes, COPD, AF, and HR	P	116
Lourenco 2019	439	220	75.5	12	All-cause mortality (continuous and categorical); cutoff: ≥ 40% increase in serum CRP	*M*	Significant variables in univariate analysis	P	36
Matsubara 2014	360	200	70.5	9.9	All-cause mortality (continuous)	*U*	N/A	P	30
Matsushita 2019	2238	1119	80 (median)	N/A	All-cause mortality (continuous)	*M*	Forward selection based on likelihood ratio	R	N/A
Otsuka 2015	96	60	69	8	1. All-cause mortality (categorical); cutoff: ≥ upper tertile 2. CV mortality (categorical); cutoff: ≥ upper tertile	*M*	Significant variables in univariate analysis	P	43
Sabatine 2007	3771	3057	63.7	8.2	1. All-cause mortality (categorical); cutoff: ≥ 3 mg/l 2. CV mortality (categorical); cutoff: ≥ 3 mg/l	*M*	Age, sex, total cholesterol, SBP, DBP, history of diabetes, smoking, BMI, history of HT, history of MI, eGFR, aspirin, *β*-blocker, and lipid-lowering therapy	P	58
Sanders-van Wijk 2015	570	345	76.8	7	All-cause mortality	*U*	N/A	R	18
Sugano 2018	191	99	76.4	11.9	1. All-cause mortality (continuous) 2. CV mortality (continuous)	*U*	N/A	P	12
Vrslaović 2015	319	212	71 (median)	N/A	Long-term CV events (categorical); cutoff: > 5 mg/l	*M*	Age, sex, traditional CV risk factors, anemia, polyvascular disease, CLI, statin treatment, and eGFR < 60 mL/min	P	24

### Diagnostic value of CRP in predicting new onset HFpEF

**Fig. 2:**
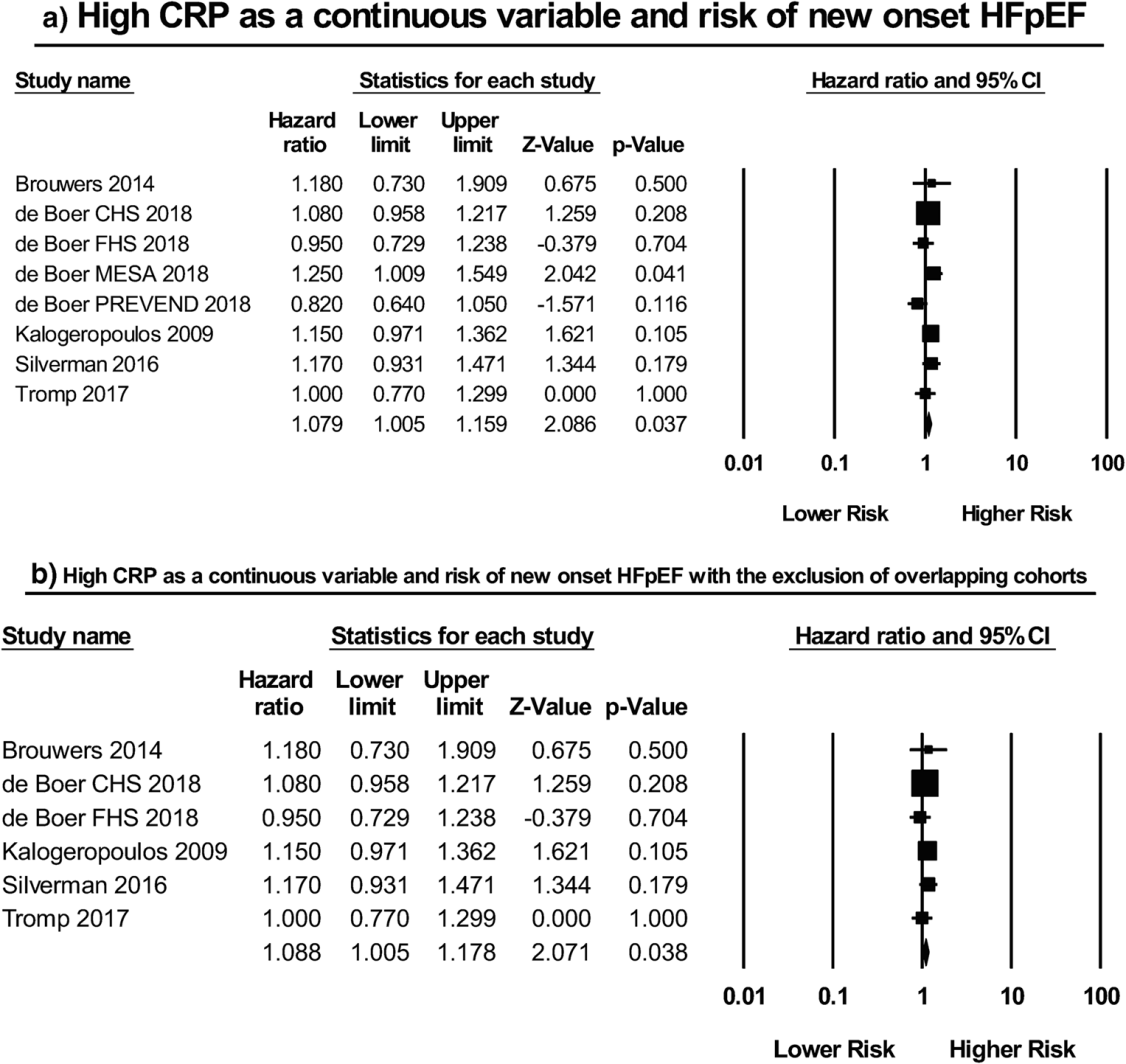
High CRP as a continuous variable and risk of new onset HFpEF: **a** without the exclusion of overlapping cohorts; **b** with the exclusion of overlapping cohorts

Eight studies [5–9] examined the correlation between CRP as a continuous variable and the risk of new onset HFpEF (Fig. 2a). Among these, De Boer et al. examined the risk of HFpEF development in four longitudinal cohorts (the Framingham Heart Study (FHS); the Cardiovascular Health Study (CHS); the Prevention of Renal and Vascular End-stage Disease (PREVEND) cohort; the Multi-Ethnic Study of Atherosclerosis (MESA)). Meta-analysis of the included studies using the independent associations reported in de Boer et al. revealed a significant relationship between CRP and HFpEF development (HR: 1.08; 95% CI: 1.01–1.16; *P* = 0.04; *I*^2^ = 22%). It should be noted that Brouwers et al. and Silverman et al. also assessed this relationship using the PREVEND and MESA cohorts, respectively, but did so using a larger sample size. As a result, in order to avoid potentially meta-analyzing overlapping populations a subset analysis was performed, with the findings of Browuers et al. (PREVEND Cohort) and Silverman et al. (MESA cohort) included and those of the PREVEND and MESA patients in de Boer et al. excluded (Fig. 2b). Subsequent meta-analysis in turn showed that a high CRP was still significantly associated with a 9% increase in the risk of new onset HFpEF, a result reported with a lower heterogeneity than that of the aforementioned (HR: 1.09; 95% CI: 1.01–1.18; *P* = 0.04; *I*^2^ = 0%).

### Prognostic value of CRP in predicting outcomes following HFpEF

#### Cardiovascular mortality

**Fig. 3:**
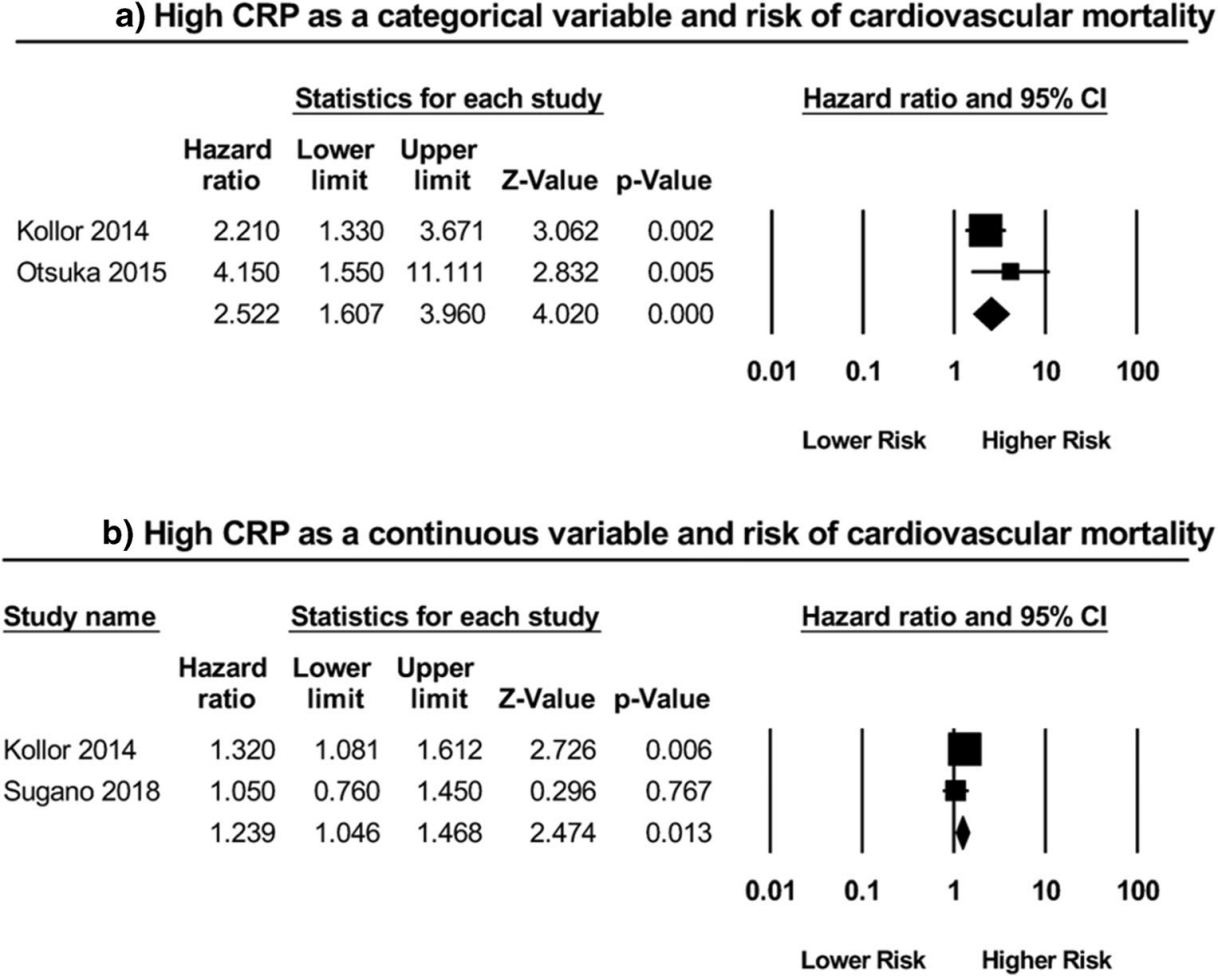
High CRP as a **a** categorical variable and risk of cardiovascular mortality; as a **b** continuous variable and risk of cardiovascular mortality

Two studies [10, 11] examined the prognostic value of CRP as a categorical variable in predicting CV mortality in HFpEF patients (Fig. 3a). Subsequent meta-analysis revealed that a high CRP significantly predicted a greater than twofold increase in the risk of CV mortality (HR: 2.52; 95% CI: 1.61–3.96; *P* < 0.0001; *I*^2^ = 19%). Furthermore, two additional studies [10, 12] also assessed this relationship but instead utilized CRP as a continuous variable (Fig. 3b). Of these two, only one reported a significant relationship. Meta-analysis of these studies indicated yet another significant, albeit smaller, association (HR: 1.24; 95% CI: 1.05–1.47; *P* = 0.01; *I*^2^ = 28%).

#### Cardiovascular outcomes

**Fig. 4:**
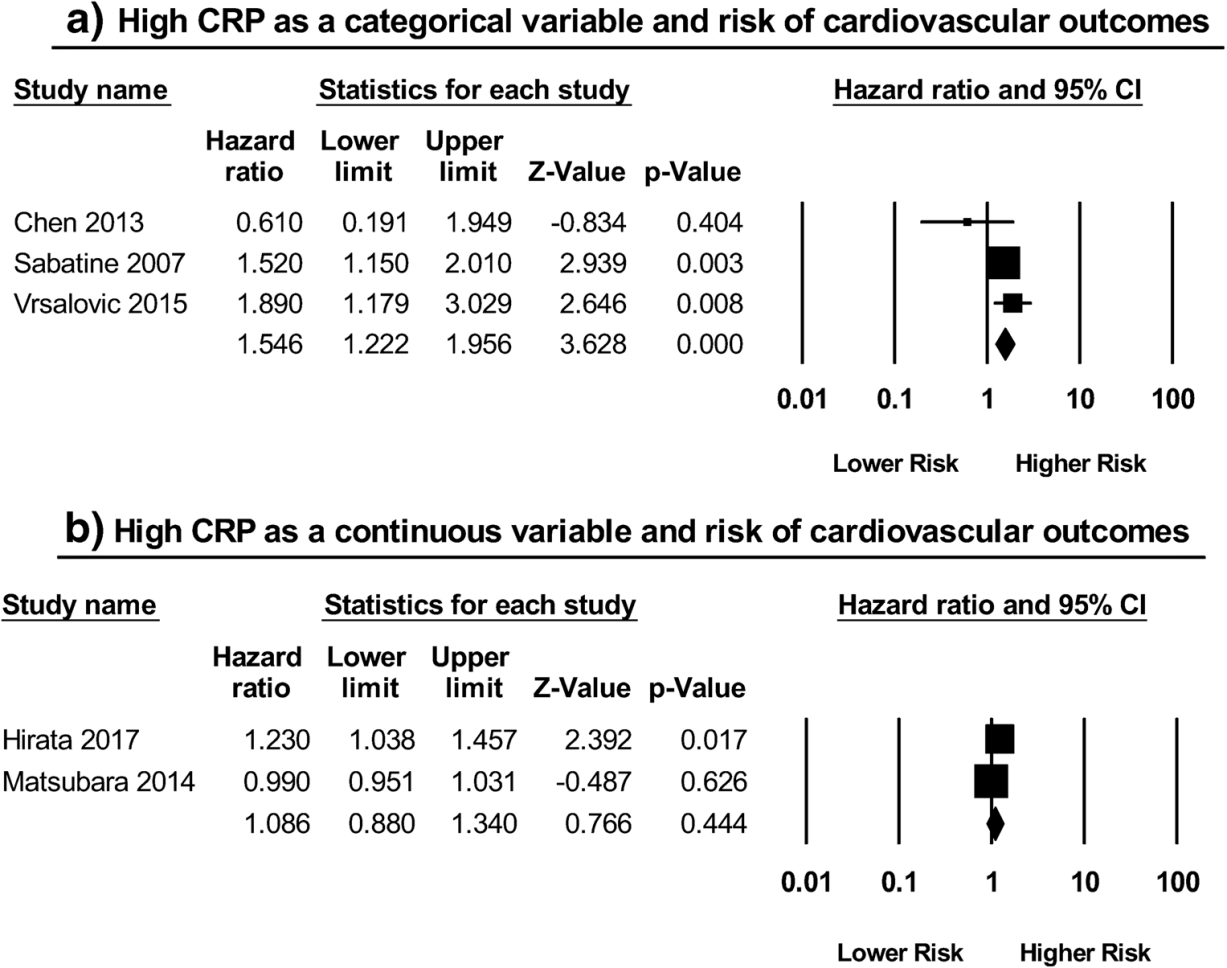
High CRP as a **a** categorical variable and risk of cardiovascular outcomes; as a **b** continuous variable and risk of cardiovascular outcomes

A total of three studies [13–15] assessed the relationship between CRP as a categorical variable and the risk of long-term adverse CV outcomes (Fig. 4a). Chen et al. used a composite endpoint of CV death, myocardial infarction (MI), and unstable angina pectoris, while Vrsalović et al. and Sabatine et al. employed composite outcomes of MACE and CV death, MI, or stroke respectively. Two of these three studies were independently significant, and subsequent meta-analysis showed that a high CRP was in fact related to a greater risk of long-term adverse cardiovascular outcomes (HR: 1.55; 95% CI: 1.22–1.96; *P* < 0.0003; *I*^2^ = 37%). Two studies [16, 17] examined such a relationship by using CRP as a continuous variable (Fig. 4b). Both employed the same composite endpoint in their investigations: CV death, MI, unstable angina pectoris, non-ischemic stroke, hospitalization for HF decompensation, and coronary revascularization. Meta-analysis of these studies, however, did not report a significant association (HR: 1.09; 95% CI: 0.88–1.34; *P* = 0.44; *I*^2^ = 83%).

#### All-cause mortality

**Fig. 5:**
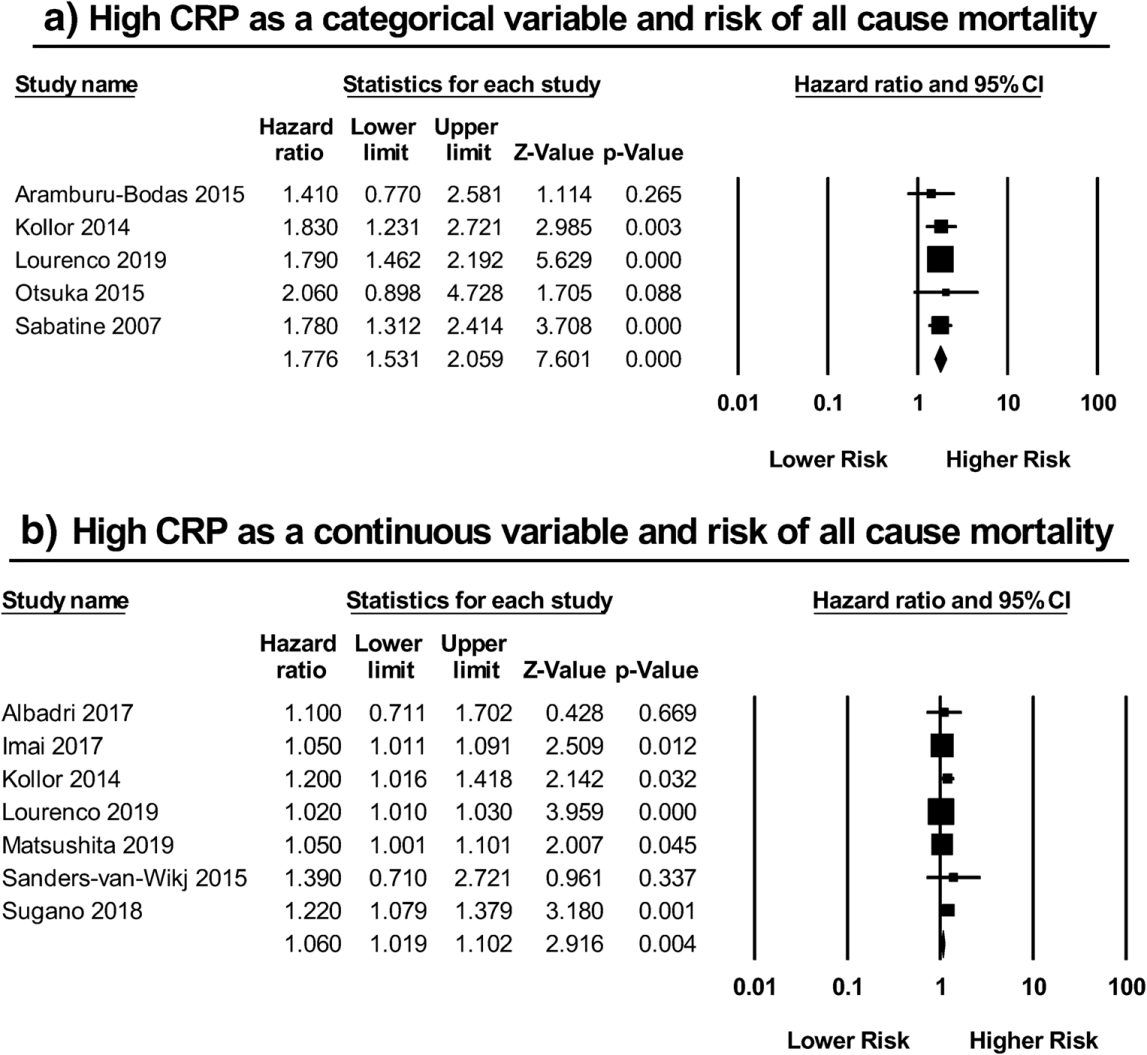
High CRP as a **a** categorical variable and risk of all-cause mortality; as a **b** continuous variable and risk of all-cause mortality

Five studies [10, 11, 14, 18, 19] investigated the correlation between CRP as a categorical variable and all-cause mortality in HFpEF patients (Fig. 5a). Meta-analysis of these studies showed that a high CRP predicted a 78% increase in the risk of all-cause mortality (HR: 1.78; 95% CI: 1.53–2.06; *P* < 0.00001; *I*^2^ = 0%). Likewise, similar findings were demonstrated when employing CRP as a continuous variable (Fig. 5b). A total of seven studies [10, 12, 19–23] assessed this relationship, of which only four reported significant results. Subsequent meta-analysis of these studies, however, illustrated yet again that a high CRP as a continuous variable was significantly associated with an increased risk of all-cause mortality (HR: 1.06; 95% CI: 1.02–1.10; *P* = 0.004; *I*^2^ = 61%).

### Sensitivity and bias analyses

The results of sensitivity analysis performed by excluding one study at a time are shown in Supplementary Figure 1A to H. The results of publication bias are shown in Supplementary Figures 2A to E.

## Discussion

The findings of this systematic review and meta-analysis are that CRP has both diagnostic value in predicting the risk of new onset HFpEF, as well as prognostic value in predicting cardiovascular outcomes and all-cause mortality following HFpEF development.

HFrEF and HFpEF are binary oppositions on the same disease spectrum. The mechanism of HFrEF is well understood, and primarily involves an initial insult that triggers myocardial injury as well as adverse remodeling, culminating in a reduction of cardiac output [24]. However, HFpEF instead represents a more complex syndrome comprised of a heterogeneous phenotype secondary to many different interacting pathophysiological processes [25]. As aforementioned, the systemic pro-inflammatory hypothesis serves as a novel paradigm for the pathogenesis of HFpEF. Paulus et al. indicates that this systemic inflammatory state is induced by multiple underlying, synergistically acting comorbidities, many of which were consistently present at baseline in most of the HFpEF cohorts included in this meta-analysis, such as obesity, hypertension, diabetes mellitus (DM), and chronic obstructive pulmonary disease (COPD) [26]. This inflammation is characterized by a patterned elevation of cytokines and acute phase reactants, which collectively mediate the production of reactive oxygen species (ROS) from the coronary endothelium, thereby leading to a decrease in nitric oxide-cyclic guanosine monophosphate (NO-cGMP) signaling in the adjacent myocardium. It should be noted that CRP not only contributes to a reduction in coronary endothelial NO bioavailability by this mechanism but also likely through its direct downregulation of endothelial nitric oxide synthase [27]. All in all, impairment of the NO-cGMP pathway in turn leads to collagen deposition, left ventricular remodeling, and subsequent diastolic dysfunction [26].

Although there is paucity in literature discussing the role of CRP in HFpEF, CRP has long known to be correlated with a higher incidence of cardiac events in patients with existing cardiovascular disease (CVD). CRP can be synthesized by both hepatic and extrahepatic tissues with the rate of its production conditioned on the severity of the underlying pathological condition [28], making it a sensitive marker for risk assessment [29]. Nonetheless, despite the demonstrated predictive value of baseline CRP in CVD [30, 31], problems exist with its use primarily due to its lack of specificity. Furthermore, currently in clinical practice, serum CRP concentrations are traditionally categorized into three tertiles: (i) < 1 mg/l, (ii) 1–3 mg/l, and (iii) > 3 mg/l, wherein tertile two and tertile three are associated with a 50% and 100% greater cardiovascular risk than tertile one, respectively [32]. However, in an observational study showcasing the effect of population characteristics on CRP, Werner et al. illustrates that age, sex, and ethnicity can all impact serum CRP concentrations. This influence of patient demographics potentially accounts for the various, distinct CRP cutoffs present in the studies included in this meta-analysis, and in turn lends credence to the notion that cutoffs adjusted for baseline factors are needed in order to achieve adequate risk stratification in the clinical setting [33].

### Limitations

There are several limitations for this systematic review and meta-analysis that should be noted. Firstly, this is a study-level meta-analysis in contrast to a data-level meta-analysis, which could improve the accuracy of our findings. Secondly, significant heterogeneity (*I*^2^ > 50) was observed with certain effect sizes. This may be due to the differences in the characteristics of the study populations, study design, CRP cutoff values or intervals, blood sampling time, and CRP measurement method (as serum hsCRP or CRP). Thirdly, as a marker for systemic inflammation, it is clearly evident that serum CRP concentrations can be affected by many diseases other than HFpEF that also predispose to a pro-inflammatory state. Some studies consisted of patients with HFpEF that developed as sequelae to such underlying inflammatory conditions including but not limited to coronary artery disease, peripheral vascular disease, end-stage renal disease, and hypertensive heart disease. In these cases, it is difficult to determine whether the elevation of CRP in these subjects is due to the independent effect of the disease itself, the HFpEF phenotype, or most likely, a combination of both. As such, the presence of co-existing diseases may have not only skewed the overall effect sizes but also, in light of its aforementioned lack of specificity, CRP will likely only be useful as an accessorial form of guidance in the clinical management of HFpEF patients, as opposed to a forerunning parameter used as the basis of risk stratification. Fourthly, it must be noted that there is likely an association between CRP and the severity of HFpEF because, as aforementioned, the degree of elevation in serum CRP concentration is related to the extent of the underlying inflammatory condition. As a result, the relationship between CRP and clinical outcomes may simply reflect a relationship between HFpEF severity and clinical outcomes. If CRP is merely a surrogate for HFpEF severity, then it is likely that CRP is a marker, rather than a predictor, for outcomes in HFpEF.

## Conclusions

The findings of this systematic review and meta-analysis indicate that CRP could be used as a biomarker that not only predicts the development of new onset HFpEF but also clinical outcomes following HFpEF in the long run. However, it should be noted that the present study possesses all the traditional limitations that typically accompany meta-analyses, and as such, readers should consider the fact that the reported diagnostic and prognostic potential of CRP in HFpEF is only accurate if said limitations are of no relevance. As a result, given the inherent methodological restrictions of this analysis, further prospective studies are still needed to not only explore the utility and dynamicity of CRP in HFpEF but also to determine whether risk stratification algorithms incorporating CRP actually provide a material benefit in bettering long-term outcomes. All in all, obtaining a greater understanding of CRP in the context of HFpEF through a consistent, repetitive demonstration of such findings in the in future prospective investigations, new treatment strategies can be devised and patient guidance can accordingly be enhanced to improve overall prognosis.

## Electronic supplementary material

ESM 1 (PDF 124 kb)

ESM 2 (PNG 201 kb)

ESM 3 (PNG 213 kb)

ESM 4 (PNG 229 kb)

ESM 5 (PNG 128 kb)

ESM 6 (PNG 123 kb)

ESM 7 (PNG 65 kb)
