# Supplementary material for: Diagnostic and prognostic value of serum C-reactive protein in heart failure with preserved ejection fraction: a systematic review and meta-analysis
Source: Heart Fail Rev. 2020 Feb 6;26(5):1141–50. doi: 10.1007/s10741-020-09927-x (PMC8310477; doi:10.1007/s10741-020-09927-x)
Supplement: Supplementary file 1 — (PDF 124 kb) [file 10741_2020_9927_MOESM1_ESM.pdf]

Supplementary Table 1: NOS risk of bias scale for included cohort studies

	Selection					Outcome			
Studies	Representativeness of the exposed cohort	Selection of the non-exposed cohort	Ascertainment of exposure	Outcome of interest not present at start of study	Comparability	Assessment of outcome	Adequacy of duration of follow-up	Adequacy of completeness of follow-up	Total score (0-9)
Albadri 2017	1	0	1	1	0	1	1	1	6
Aramburu-Bodas 2015	1	1	1	1	1 (LVEF)	1	1	1	8
Brouwers 2014	1	1	1	1	0	1	1	1	7
Chen 2013	1	1	1	1	1 (LVEF)	1	1	1	8
De Boer 2018	1	1	1	1	0	1	1	1	7
Hirata 2017	1	1	1	1	1 (age)	1	1	1	8
Imai 2017	1	1	1	1	1 (LVEF)	1	1	1	8
Kalogeropoulos 2009	1	1	1	1	0	1	1	1	7
Koller 2014	1	1	1	1	2 (age, LVEF)	1	1	1	9
Lourenco 2019	1	1	1	1	0	1	1	1	7
Matsubara 2014	1	1	1	1	1 (LVEF)	1	1	1	8
Matsushita 2019	1	1	1	1	2 (age, LVEF)	1	1	1	9
Otsuka 2018	1	1	1	1	1 (LVEF)	1	1	1	8
Sabatine 2007	1	1	1	1	2 (age, LVEF)	1	1	1	9
Sanders 2015	1	1	1	1	0	1	1	1	7
Silverman 2016	1	1	1	1	0	1	1	1	7
Sugano 2018	1	1	1	1	2 (age, LVEF)	1	1	1	9
Tromp 2017	1	1	1	0	1 (LVEF)	1	1	1	7
Vrsalović 2015	1	0	1	1	0	1	1	1	6

LVEF = left ventricular ejection fraction
