# Supplementary figures and images for: Diagnostic and prognostic value of serum C-reactive protein in heart failure with preserved ejection fraction: a systematic review and meta-analysis
Source: Heart Fail Rev. 2020 Feb 6;26(5):1141–50. doi: 10.1007/s10741-020-09927-x (PMC8310477; doi:10.1007/s10741-020-09927-x)

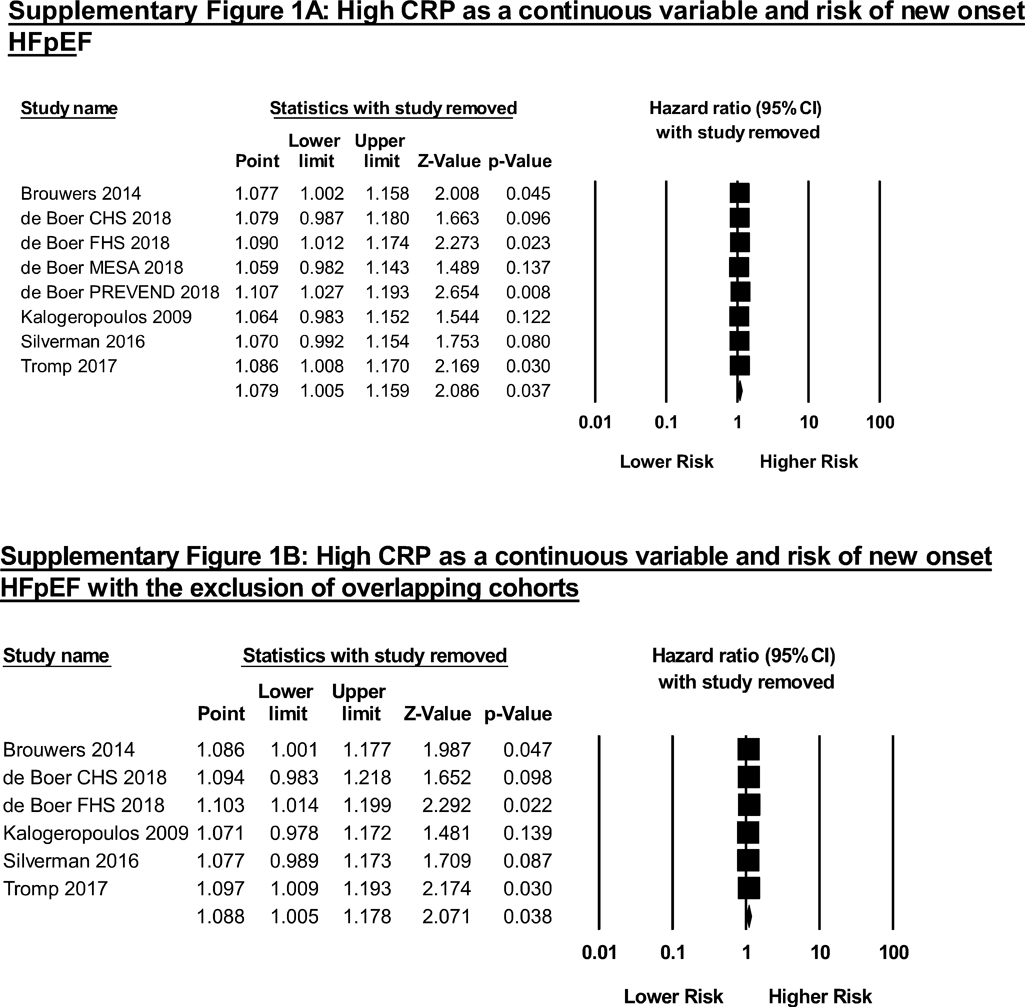

Supplement: Supplementary file 2 — (PNG 201 kb) [file 10741_2020_9927_MOESM2_ESM.png]

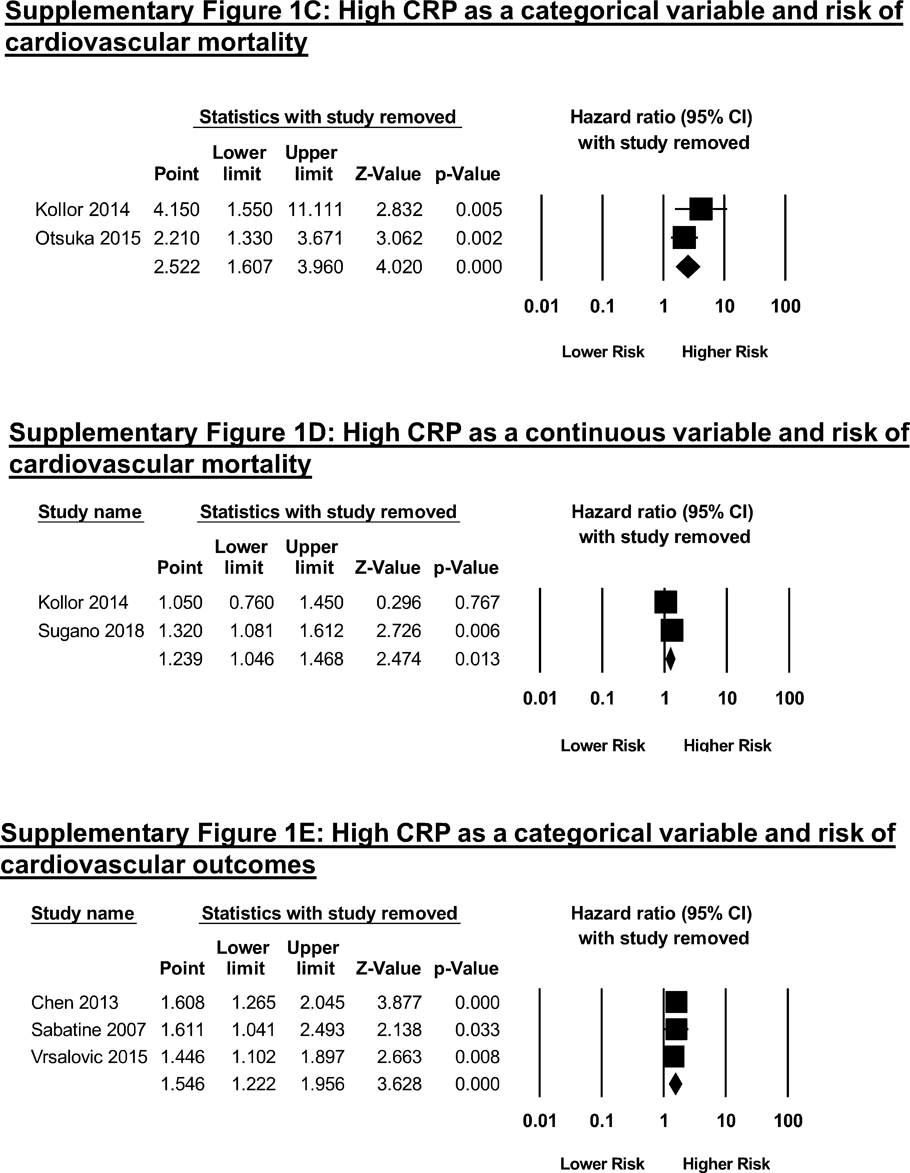

Supplement: Supplementary file 3 — (PNG 213 kb) [file 10741_2020_9927_MOESM3_ESM.png]

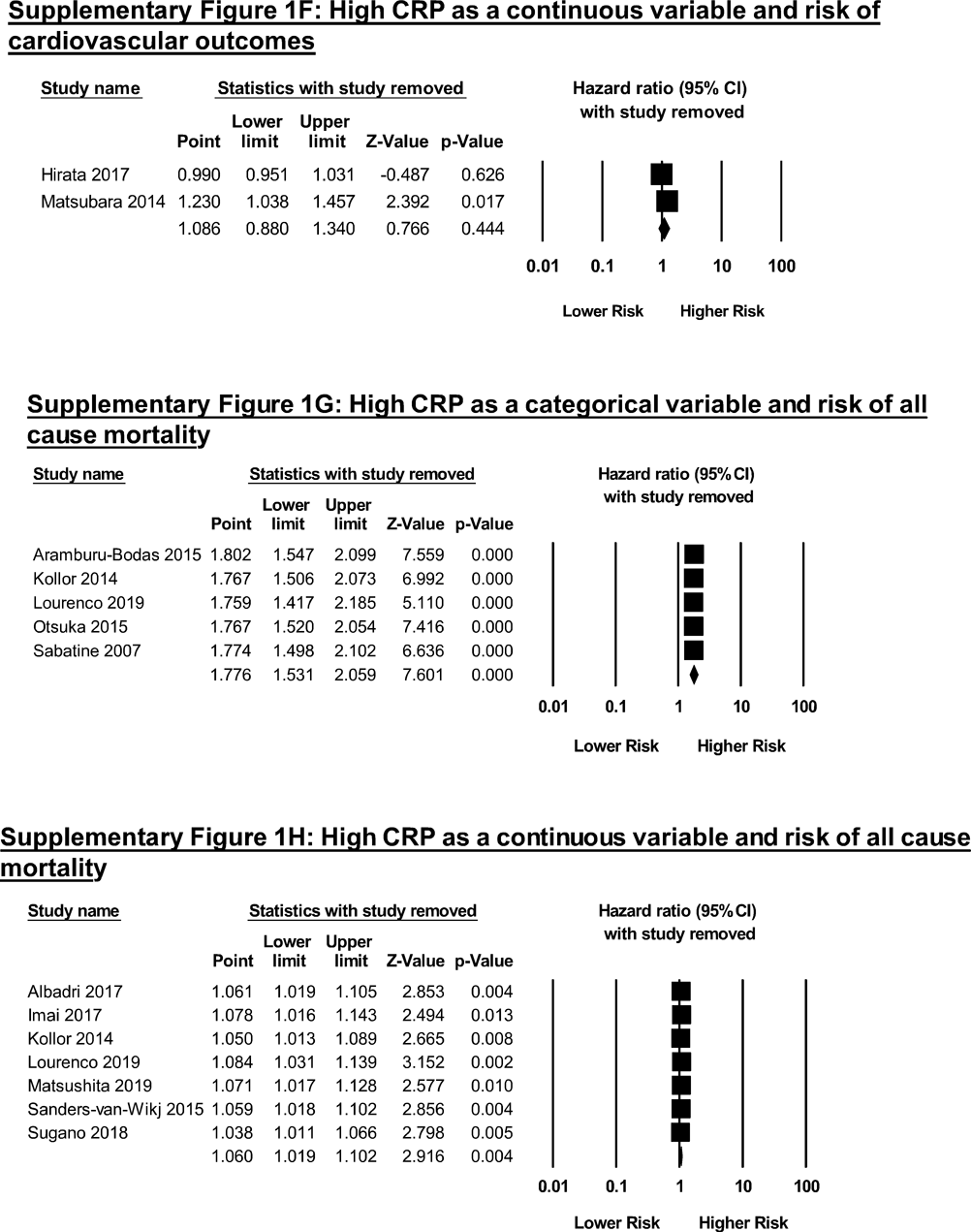

Supplement: Supplementary file 4 — (PNG 229 kb) [file 10741_2020_9927_MOESM4_ESM.png]

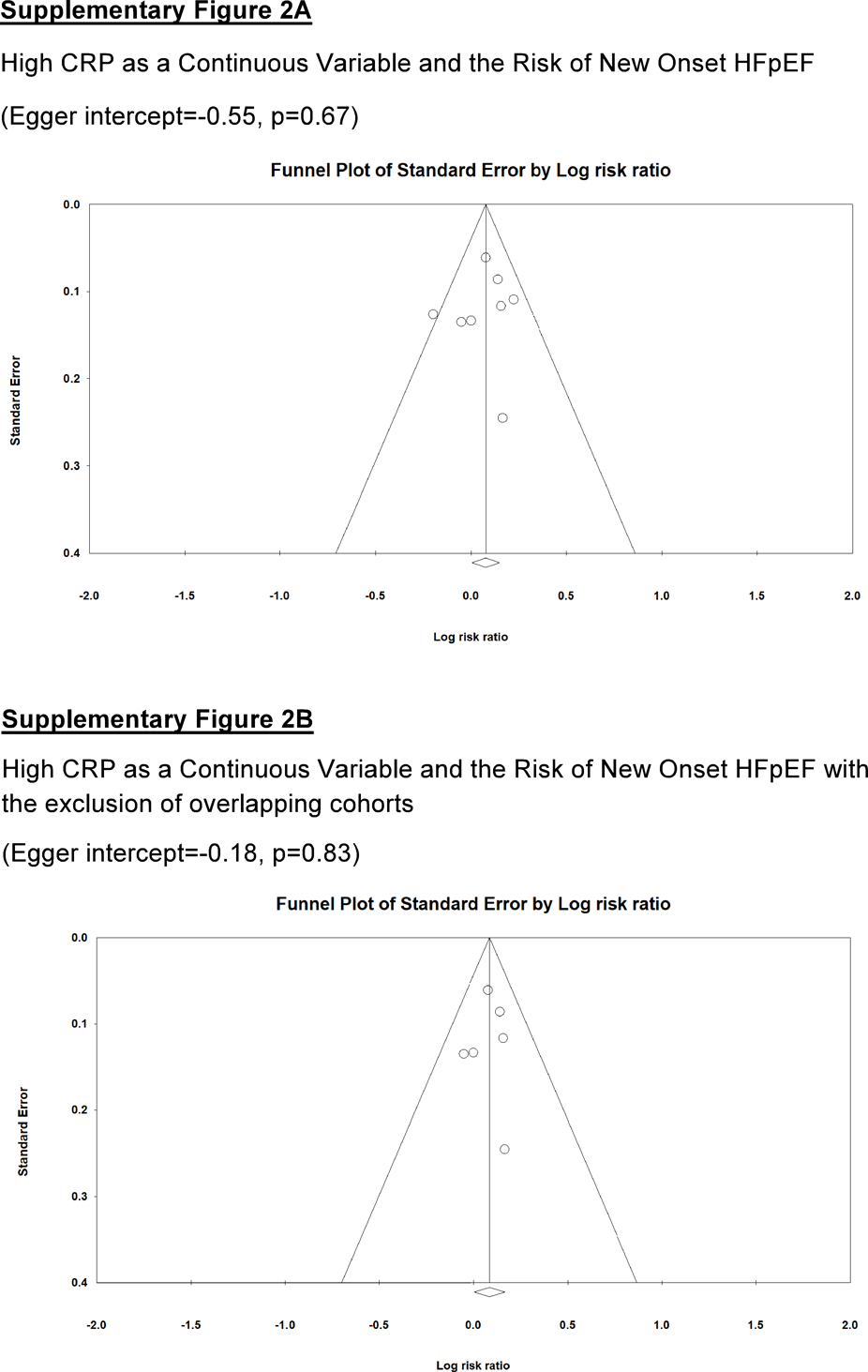

Supplement: Supplementary file 5 — (PNG 128 kb) [file 10741_2020_9927_MOESM5_ESM.png]

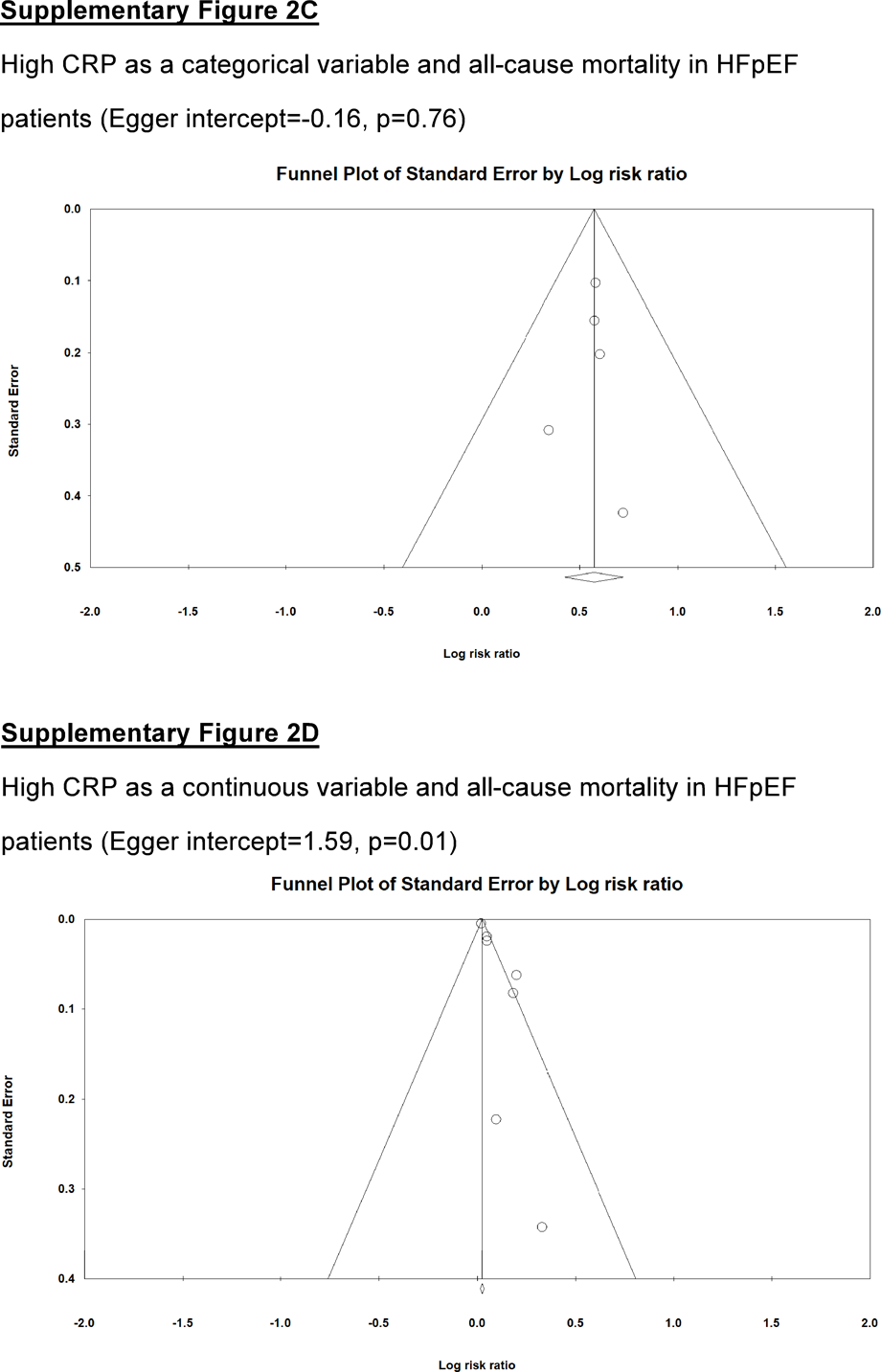

Supplement: Supplementary file 6 — (PNG 123 kb) [file 10741_2020_9927_MOESM6_ESM.png]

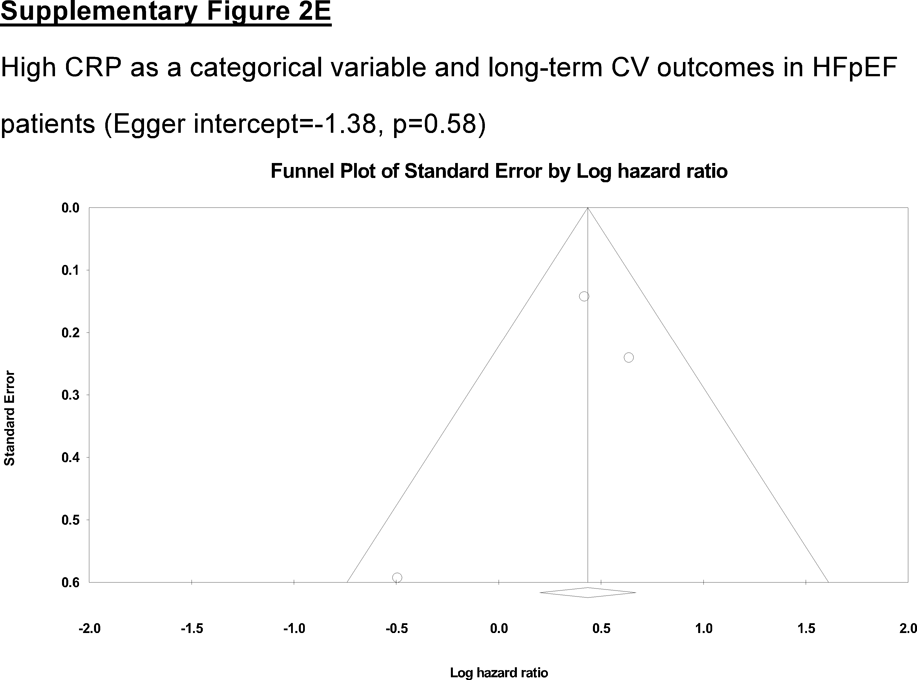

Supplement: Supplementary file 7 — (PNG 65 kb) [file 10741_2020_9927_MOESM7_ESM.png]
